# The role of versican isoforms V0/V1 in glioma migration mediated by transforming growth factor-*β*2

**DOI:** 10.1038/sj.bjc.6603766

**Published:** 2007-04-24

**Authors:** F Arslan, A-K Bosserhoff, T Nickl-Jockschat, A Doerfelt, U Bogdahn, P Hau

**Affiliations:** 1 Department of Neurology, University of Regensburg, Universitaetsstrasse 84, Regensburg 93053, Germany; 2 Institute of Basic Medical Sciences, University of Oslo, Sognsvannsveien 9, Oslo 0317, Norway; 3 Institute of Pathology, University of Regensburg, Franz-Josef-Strauß-Allee 11, Regensburg 93053, Germany; 4 Department of Psychiatry and Psychotherapy, RWTH Aachen University, Pauwelsstrasse 30, Aachen 52074, Germany

**Keywords:** Versican, TGF-*β*, high-grade glioma, migration

## Abstract

Versican is a large chondroitin sulphate proteoglycan produced by several tumour cell types, including high-grade glioma. The increased expression of certain versican isoforms in the extracellular matrix (ECM) plays a role in tumour cell growth, adhesion and migration. Transforming growth factor-*β*2 (TGF-*β*2) is an important modulator of glioma invasion, partially by remodeling the ECM. However, it is unknown whether it interacts with versican during malignant progression of glioma cells. Here, we analysed the effect of TGF-*β*2 on the expression of versican isoforms. The expression of versican V0/V1 was upregulated by TGF-*β*2 detected by quantitative polymerase chain reaction and immunoprecipitation, whereas V2 was not induced. Using time-lapse scratch and spheroid migration assays, we observed that the glioma migration rate is significantly increased by exogenous TGF-*β*2 and inhibited by TGF-*β*2-specific antisense oligonucleotides. Interestingly, an antibody specific for the DPEAAE region of glycosaminoglycan-*β* domain of versican was able to reverse the effect of TGF-*β*2 on glioma migration in a dose-dependent manner. Taken together, we report here that TGF-*β*2 triggers the malignant phenotype of high-grade gliomas by induction of migration, and that this effect is, at least in part, mediated by versican V0/V1.

Glioma cell invasion is a complex and multi-step mechanism involving a large array of molecules and cell–cell and cell–extracellular matrix (ECM) interactions. These processes allow individual tumour cells to migrate into and invade the healthy surrounding brain even after surgical resection, leading to the failure of current therapeutic modalities (Goldbrunner *et al*, 1998; Bellail *et al*, 2004).

Transforming growth factor-*β* (TGF-*β*) is a multifunctional cytokine which interferes with immune responses and which modulates migration, invasion and angiogenesis in high-grade glioma. In this regard, TGF-*β* antagonistic strategies are among the most promising of the current innovative approaches targeting glioblastoma, particularly in conjunction with novel approaches of immunotherapy and vaccination (Wick *et al*, 2006).

TGF-*β* exerts a complex set of effects in cancers. In early stages of tumour development, it inhibits tumour growth, but later on, it turns to a highly tumorigenic molecule, including increased tumour cell motility and invasion, induction of angiogenesis and immune suppression. The three different isoforms of TGF-*β* (TGF-*β*1, TGF-*β*2, TGF-*β*3) are differentially expressed in high-grade glioma (Kjellman *et al*, 2000).

The importance of TGF-*β*1 decreases with the tumour grade in high-grade gliomas (Jachimczak *et al*, 1996; Pan *et al*, 2006), and its expression does not correlate to time to progression (Hau *et al*, 2006). However, some authors report on TGF-*β*1-stimulated migration and invasion of glioma cells (Merzak *et al*, 1994; Platten *et al*, 2000), and marked inhibition of glioma invasion modulated by TGF-*β*1-specific antisense oligonucleotides (Paulus *et al*, 1995) and by RNA interference targeting both TGF-*β*1 and TGF-*β*2 was reported (Friese *et al*, 2004).

In comparison to TGF-*β*1 and TGF-*β*3, TGF-*β*2 is the predominant isoform of TGF-*β* secreted by human malignant glioma cells (Kjellman *et al*, 2000). TGF-*β*2-targeted therapies are currently evaluated in randomised clinical trials (Fakhrai *et al*., 2006; Schlingensiepen *et al*, 2006) in consistent with the reports on the relevance of TGF-*β*2 for the progression of high-grade gliomas. TGF-*β*2-derived immunosuppression of glioma patients is well described (Jachimczak *et al*, 1993; Grauer *et al*, 2006) and plays a pivotal role in glioma progression. In addition, there is increasing evidence for a prominent role of TGF-*β*2 in glioma cell motility (Platten *et al*, 2001; Uhl *et al*, 2004). TGF-*β*-triggered glioma cell motility is based on a very complex system consisting of a step-like process of attachment and migration, which involves components of ECM, proteases and integrins as well as the tumour cells (Platten *et al*, 2001). Understanding the functions and regulatory processes of glioma cell migration is critical for developing appropriate anti-invasive therapies.

Versican, a large multi-domain chondroitin sulfate (CS) proteoglycan, is a major component of the ECM involved in cell adhesion, migration, proliferation and differentiation, all processes vital to tumour development and progression (Landolt *et al*, 1995; Zhang *et al*, 1998; Ang *et al*, 1999; Cattaruzza *et al*, 2004). Versican consists of an N-terminal globular domain (G1), a selectin-like domain (G3) at the C-terminal and the central glycosaminoglycan (GAG) attachment domains, which are encoded by exons that can undergo differential splicing (Lemire *et al*, 1999). Alternative splicing of versican generates at least four isoforms known as V0, which contains both GAG-*α* and GAG-*β* exons; V1, containing the GAG-*β* exon; V2, having the GAG-*α* exon and V3, consisting only of the globular domains. Versican isoforms V0/V1 are mainly expressed in the late stages of embryonic development (Landolt *et al*, 1995), whereas versican V2 is the predominant CS proteoglycan in the mature brain (Schmalfeldt *et al*, 2000). The isoforms play distinct roles due to a difference in CS domains (Sheng *et al*, 2005).

Versican has been reported to be upregulated by TGF-*β* in a variety of cells (Kahari *et al*, 1991; Schonherr *et al*, 1991; Robbins *et al*, 1997; Venkatesan *et al*, 2002; Zhao and Russell, 2005). We have previously found that TGF-*β*2-specific phosphorothioate antisense molecules inhibit glioma migration in migration assays and downregulate versican expression in gene arrays (Nickl-Jockschat *et al*, 2007). Adhering to these previous results, we investigated the distinct biological roles of versican isoforms in the tumorigenesis of high-grade gliomas to determine whether the major ECM proteoglycan versican plays a role in TGF-*β*2-mediated migration of glioma cells leading to malignant progression of human high-grade gliomas.

## MATERIALS AND METHODS

### Cell culture

Different types of glioma cell lines and primary cultures were used for *in vitro* experiments. Human high-grade glioma cell lines U87MG and A172 were obtained from American Type Culture Collection (Manassas, VA, USA). The gliomas named as ‘HTZ’ were primary tumour cell cultures derived from surgical specimens of human high-grade gliomas (WHO Grade IV) as described before (Bogdahn *et al*, 1989). All tumour cells were maintained as standard monolayer cultures in tumour growth medium at 37°C, 5% CO_2_, 95% humidity in a standard tissue culture incubator. Growth medium was comprised of Dulbecco's modified Eagle's medium (Invitrogen, Carlsbad, CA, USA) supplemented with 10% fetal calf serum (FCS).

To elucidate the effect of exogenous TGF-*β*2 on the regulation of versican isoforms, we performed treatment assays with different concentrations of TGF-*β*2; glioma cells were seeded at an equal density in cell culture flasks containing growth medium as described above. After 24 h, triplicates of subconfluent cells were treated with four different concentrations (1, 5, 10 and 50 ng ml^−1^) of activated rhTGF-*β*2 protein (R&D Systems, Minneapolis, MN, USA) and incubated for 72 h. Cells and supernatants were harvested to prepare mRNA or protein as described below. In time-point assays, cells were treated with 10 ng ml^−1^ of TGF-*β*2 and harvested at three different time points: days 1, 3 and 5.

### TGF-*β*2-specific antisense phosphorothioate oligodeoxynucleotides

The TGF-*β*2-specific antisense phosphorothioate oligodeoxynucleotides (PTOs) (AS-11) as described previously (Nickl-Jockschat *et al*, 2007) was used with the sequence 5′-GTAGTGCATTTTTTAAAAAA-3′ (mRNA target region 171–190) (Sigma-Genosys, Steinheim, Germany). As a control PTO, we used NS (mis) with three mismatch bases (sequence: 5′-GTAATGAATGTTTTAAAAAA-3′).

### Reverse transcriptase polymerase chain reaction

Total RNA was extracted from tumour cells with the RNA purification system RNeasy Mini Kit (Qiagen, Hilden, Germany) following the manufacturer's instructions. RNA concentration and purity was determined by measuring optical density at wavelengths of 260 and 280 nm using a standard spectrophotometer. First-strand cDNA generated from 1 *μ*g of total RNA samples by using a reverse transcription kit (Promega, Madison, WI) was used to amplify gene-specific cDNAs from expressed genes. Appropriate forward and reverse primers to detect transcripts of interest were used in reverse transcriptase polymerase chain reaction (RT–PCR) reactions for cDNA amplification. The primers used were as follows: TGF-*β*2 (forward: 5′-TCTAGGGTGGAAATGGATACACGAACC-3′; reverse: 5′-TGTTACAAGCATCATCGTTGTCGTCG-3′) resulting in a 314 bp fragment. Versican primers (forward: 5′-GTGACTATGGCTGGCACAAATTCC-3′; reverse: 5′-GGTTGGGTCTCCAATTCTCGTATTGC-3′) were designed to detect all known splice variants of the gene resulting in 229 bp fragment.

The specific primers for the PCR amplification of versican isoforms were used as described before (Cattaruzza *et al*, 2002). The sizes of the amplification products were 405 bp for the V0 splice form (forward: 5′-TCAACATCTCATGTTCCTCCC-3′ and reverse: 5′-TTCTTCACTGTG GGTATAGGTCTA-3′), 336 bp for V1 (forward: 5′-GGCTTTGACCAGTGCGATTAC-3′; reverse: 5′-TTCTTCACTGTGGGTATAGGTCTA-3′), 498 bp for V2 (forward: 5′-TCAACA TCTCATGTTCCTCCC-3′; reverse: 5′-CCAGCCATAGTCACA TGTCTC-3′) and 429 bp for V3 (forward: 5′-GGCTTTGACCAGTGCGATTAC-3′; reverse: 5′-CCAGCCATAGTCACATGTCTC-3′). Annealing temperatures were optimised for each primer pair using the following program: DNA polymerase was activated at 95°C for 5 min, amplified for 30 cycles (95°C for 45 s, 57–60°C for 1 min, 72°C for 45 s) and extended at 72°C for 5 min. RT–PCR products were analysed on 1% agarose gel and visualised with ethidium bromide staining. The housekeeping gene *β*-actin was used as a positive control to assess cDNA quality.

### Quantitative PCR

To precisely quantify mRNA expression, a real-time PCR system (ABI PRISM 7000 Sequence Detection System, CA, USA) that measures nucleic acid molecules based on the detection of a fluorescent reporter molecule (SYBR Green dye) was used. Target-cDNA-specific primers as described above were established. Briefly, five serial twofold dilutions of cDNA were amplified in triplicates to construct standard curves for both the target gene and the endogenous reference (*β*-actin). Standard curves generated by the software were used for extrapolation of expression levels for the unknown samples based on their threshold cycle (*C*_T_) values. All amplifications of unknown samples were in the linear range. For each reaction melting curves and agarose gel electrophoresis of PCR products were used to verify the identity of the amplification products. Each probe was run in parallel with primers specific for *β*-actin as standard for quantification of target cDNA. The target gene amount was divided by the housekeeping gene amount to obtain a normalised target value. Each of the experimental normalised values was divided by the normalised control (untreated) sample value to generate the relative expression levels in fold.

### Immunoprecipitation

Total cell lysates were prepared in radioimmuno precipitation assay lysis buffer (20 mM Tris, pH 7.4, 150 mM NaCl, 1% Triton X-100, 0.5% sodium deoxycholate, 0.1% sodium dodecyl sulfate (SDS)), supplemented with Complete Protease Inhibitor Cocktail Tablets (Roche Molecular Biochemicals, Manheim, Germany). Equal amounts of total protein quantified in a Bicinchoninic acid (BCA) assay (Uptima, Montpellier, France) were incubated with protein G beads at 4°C for 6 h for pre-clearing. After centrifugation, the nonspecifically bound G beads were discarded and then supernatants were incubated with 2 *μ*g ml^−1^ versican V0/V1 Neo-rabit polyclonal antibody (ABR, Golden, CO) and incubated at 4°C overnight. After washing, the beads were boiled in 1 × protein loading dye for 5 min and loaded directly into pre-poured Tris-HCl-glycine SDS–polyacrylamide gel electrophoresis (PAGE) gels (10%) along with pre-stained molecular weight standards (Bio-rad Laboratories, Palo Alto, CA, USA). Electrophoresis was performed in Tris/glycine/SDS running buffer (Biorad Laboratories) at 125–150 V for a suitable migration period. Following transfer to polyvinylidene fluoride (PVDF) membranes (Biorad Laboratories) at 120 mA constant current for 1–2 h, blots were briefly washed in Tris buffered saline with Tween (TBST) (10 mM Tris, 150 mM NaCl, and 0.5% Tween-20, pH 8.0), blocked for 1 h at RT with 5% non-fat dry milk and then incubated with 1 *μ*g ml^−1^ of the versican V0/V1 Neo-antibody at 4°C overnight. Immunocomplexes were visualised using a horsedish peroxidase-conjugated antibody followed by chemoluminescence reagent (Pierce Biotechnology, Rockford, IL, USA) detection on photographic film.

### Spheroid assay

Multi-cell tumour spheroids were initiated by seeding (3–8) × 10^6^ cells incubated in agar-coated wells in order to inhibit adhesion. Mature spheroids with a mean diameter of 200–250 *μ*m were explanted to uncoated 96-well plates containing the corresponding treatment (TGF-*β*2, 10 ng ml^−1^; versican V0/V1 Neo-antibody, 2–20 *μ*g ml^−1^; AS-11, 20 *μ*M). Six spheroids were used for each experimental condition in each experiment. Spheroids were allowed to migrate for 5–7 days. Spreading of the spheroids was monitored by microscopic photographs of each spheroid after 0, 1, 3, 5 and 7 days. For quantification, the mean diameter of randomly selected glioma cells that had migrated from the tumour spheroid was measured by a blinded investigator and expressed in relation to the mean radial distance at time 0 h. Bovine serum albumin (BSA) was used as a control protein. As unrelated controls, normal rabit immunoglobulin G (IgG) (R&D Systems, Minneapolis, MN) and NS (mis) were used at the same concentrations, respectively. Assays were repeated at least twice.

After migration of cells from the spheroids, the spheroids were collected with a pipette tip. Total cell lysates of migrated cells and spheroids were prepared separately for protein expression. For versican expression, western blotting with versican V0/V1-specific antibody was used as described above.

### Scratch migration assay

The spreading and migration capabilities of HTZ-349 cells were assessed using a scratch (wound) assay measuring the expansion of a cell population on a given surface. The cells were seeded into uncoated six-well tissue culture dishes at a density of 2.5 × 10^5^ cells and cultured in medium containing 10% FCS to nearly confluent cell monolayers, which were then scratched using 1 ml sterile pipette tips. Any cellular debris was removed by washing with PBS. The wounded monolayers were then incubated in 10% FCS medium containing TGF-*β*2 (20 ng ml^−1^) and BSA (20 ng ml^−1^) as control for 48–72 h. The wound area in a marked field of view was inspected at different time intervals subsequently until closure to determine the distance migrated by the cells.

The wound areas were photographed under a light microscope by a blinded investigator. The width of the scratch was measured at 0 and 24, 48, 72 h after treatment to measure the distance traveled by the cells. The difference between the width of scratch (wound area) at 0 h and at a given time point represented the distance migrated by the cells. The quantification of the distance migrated by the cells expressed as percentages and comparison made with untreated control using statistical analysis. The experiments were repeated in duplicate wells at least three times.

### Statistics

The results (mean value+standard deviation) of control (untreated) *vs* stimulated (treated) cell samples were analysed using the Student's *t*-test for migration assays. The level of significance was set at ^*^P<0.05.

## RESULTS

### Expression profiles of TGF-*β*2 and versican isoforms in human high-grade glioma cells

**Figure 1:**
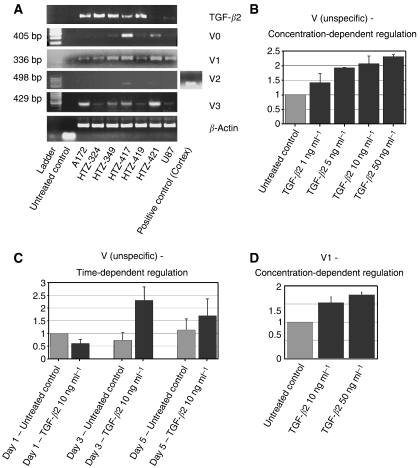
Expression of versican and regulation by TGF-*β*2. TGF-*β*2 is expressed in high-grade gliomas and upregulates versican expression in a concentration and time-dependent manner. Results show mRNA expression levels detected by PCR and qPCR. (**A**) Differential expression of versican isoforms in glioma cells. RT–PCR analysis shows the semiquantitative mRNA expression of TGF-*β*2 and versican isoforms in different human glioma cell lines. V1 was found to be expressed in all glioma cells. V2 was only detected in HTZ-417; cerebral cortex was used as a positive control for V2. V0 and V3 had a heterogeneous expression pattern. The housekeeping gene *β*-actin was used to adjust for cDNA quantity. (**B**) Regulation of versican mRNA expression in TGF-*β*2-treated HTZ-349 cells with four different concentrations (1, 5, 10, 50 ng ml^−1^). Versican expression was upregulated in a concentration-dependent manner reaching a peak of 2.3-fold increase with 50 ng ml^−1^ of TGF-*β*2. (**C**) qPCR results of versican expression in TGF-*β*2 (10 ng ml^−1^)-treated HTZ-349 cells at three different time points (days 1, 3, 5). The upregulation of versican expression was most pronounced at 72 h (2.2-fold) and then showed a trend to decrease gradually over time. (**D**) Expression of versican isoform V1 by qPCR at mRNA level detected with a specific primer in HTZ-349 cells treated with TGF-*β*2 (10, 50 ng ml^−1^). The increase of versican after treatment with TGF-*β*2 is a V1-specific effect. Normalized values with the housekeeping gene *β*-actin are reported as relative expression in folds. Mean values±standard deviations are representative of triplicates. Mean values of the untreated (control) group were set to a value of 1.

To evaluate the profiles of TGF-*β*2 and versican in two human glioma cell lines (U87, A172) and in five primary cell cultures (HTZ-324, HTZ-349, HTZ-417, HTZ-419, HTZ-421), expression levels of TGF-*β*2 and versican isoforms were detected by RT–PCR at mRNA level using *β*-actin as control gene (Figure 1A). All cell lines expressed TGF-*β*2 in different amounts. Versican isoforms were found to be differentially expressed in high-grade glioma cells; among the four isoforms, V1 was the most prominent one and found to be expressed in all glioma cells. V0 was intermediately expressed in almost 30% of glioma cell lines. V3 had a heterogeneous expression pattern. The V2 isoform was detected as a faint signal only in HTZ-417 (Figure 1A).

### Regulation of versican expression by exogenous TGF-*β*2

Next, the modulation of versican expression by TGF-*β*2 was assessed. Treatment assays were performed with the glioma cell lines U87, A172, HTZ-417 and HTZ-349. These cell lines were chosen because they secrete different levels of TGF-*β*2 and also their growth characteristics provided consistent confluency in a short period of time.

First, we analysed versican expression at the mRNA level by quantitative PCR (qPCR) with a primer binding to the G3 domain which detects all isoforms. In these experiments, a significant increase of versican expression was detected in HTZ-349 cells treated with TGF-*β*2 in comparison to untreated cells, reaching a fold level of 2.3 with a maximum dose of TGF-*β*2 (50 ng ml^−1^) (Figure 1B). Results with the other cell lines tested (U87, A172 and HTZ-417) were similar with a 1.9-, 1.7- and 2.1-fold increase, respectively (data not shown). In time-dependent assays, the upregulation of versican expression reached a peak after 72 h (2.2-fold) and then decreased gradually over time (Figure 1C).

Since versican V1 and V0 isoforms predominantly increase in tumours of different origin, suggesting that these isoforms are mainly involved in tumour development, we also elucidated whether TGF-*β*2 differentially modulates the expression of versican isoforms in high-grade glioma. We found that V1 was the most upregulated isoform with a 1.7-fold increase (Figure 1D). V0 was intermediately increased and the expression of V2 was not induced by TGF-*β*2 (data not shown).

**Figure 2:**
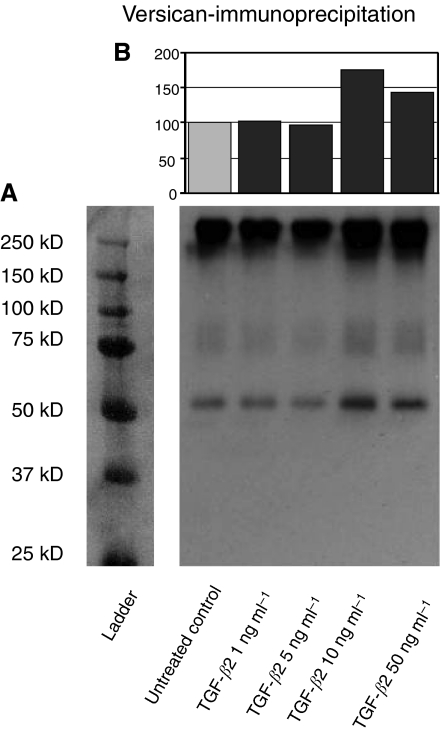
Expression of versican isoforms V0/V1 induced by TGF-*β*2 at protein level. (**A**) IP results showing the expression of versican V0/V1 in HTZ-349 cells treated with TGF-*β*2 (1, 5, 10, 50 ng ml^−1^) at protein level. An antibody specific for the DPEAAE peptide segment in the GAG-*β* domain of versican V0 and V1 was used. The major very high molecular mass products, which have the characteristic smear of proteoglycans and migrate in the 250–300 kDa range represent intact versican V1. The products migrating around 75 and 50 kDa represent the G1-DPEAAE cleavage products of human versican V1. (**B**) Densitometric analysis of the versican V0/V1 signals at 50 kDa level are presented in percentages and values of the untreated (control) group were set to 100% (upper panel). Both the intact versican V1 and cleavage products that reacted with anti-DPEAAE show upregulation, especially observed with 10 and 50 ng ml^−1^ of TGF-*β*2.

Next, we examined the expression of versican at protein level with western blot and immunoprecipitation (IP) using a polyclonal antibody (Figure 2). The versican V0/V1 Neo-antibody (Affinity BioReagents, Golden, CO, USA) is a rabbit polyclonal antibody to the versican Asp-Pro-Glu-Ala-Ala-Glu (DPEAAE) neo-epitope at the GAG-*β* domain of human versican and recognises the N-terminal (G1 domain) neo-epitope cleavage products of versican after cleavage with ADAMTS at the Glu441–Ala442 bond in the V1 isoform; the corresponding peptide bond is Glu1428–Ala1429 in the V0 isoform (Sandy *et al*, 2001). As the antibody does not react with the DPEAAE sequence when it is present in intact versican (V0) (Sandy *et al*, 2001; Kern *et al*, 2007), no high molecular mass band representing intact versican (V0), which migrates at 350–400 kDa (data not shown), could be detected in low-density gels (4%) and after chondroitinase ABC digestion. However, V1, which runs around 280 kDa, could be detected with high versican V1 concentrations (Russell *et al*, 2003; Kern *et al*, 2006).

In IP without enzymatic digestion of chondroitin sulphate chains, the products had the characteristic smear of proteoglycans to which GAG chains of different sizes have been covalently attached as described before (Yang *et al*, 1999). We detected high molecular mass intact versican V1 at around 300 kDa, which is the expected molecular mass of V1 without chondroitinase digestion in glioma cell lines (Dours-Zimmermann and Zimmermann, 1994) verifying that the antibody reacts with the DPEAAE sequence in intact versican V1 (Figure 2A). The products migrating around 75 and 50 kDa represent the G1-DPEAAE-cleaved products of human versican V1 by ADAMTS-1 and ADAMTS-4 as described before with the same antibody (Sandy *et al*, 2001; Russell *et al*, 2003; Somerville *et al*, 2003; Kern *et al*, 2006). These signals representing intact versican V1 and its cleavage products were upregulated with exogenous TGF-*β*2 in a concentration-dependent manner. The highest signals were observed with 10 and 50 ng ml^−1^ of TGF-*β*2, consistent with qPCR results. Densitometric analysis also allowed quantification of this upregulation, which showed almost 80% increase compared to untreated control (Figure 2B). Taken together, these results demonstrate that exogenous TGF-*β*2 induces not only versican V1 expression, but also cleavage of versican V1 via ADAMTS-1 and ADAMTS-4.

### Effect of TGF-*β*2 on migration of glioma cells

**Figure 3:**
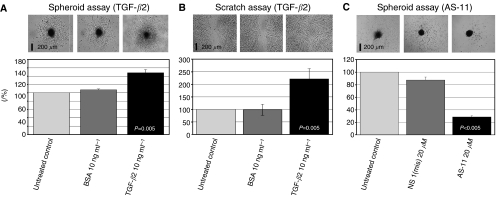
Promotion of glioma migration by TGF-*β*2. (**A**) The effect of exogenous TGF-*β*2 on glioma migration was analysed in HTZ-349 spheroid assays. The spheroids were allowed to migrate in absence or presence of TGF-*β*2 (20 ng ml^−1^) for 5 days. BSA (20 ng ml^−1^) was used as a protein control. Results are the mean±standard deviation of two experiments and six spheroids used for each experimental condition. Migration areas measured as described in methods are represented in percentages. Untreated controls were set to 100%. Exogenous TGF-*β*2 significantly induced migration compared to untreated control and BSA (^**^P<0.01). Images taken from each experimental condition at day 5 are shown in the upper panel. The scale bar is 200 *μ*m. *P*-values (untreated *vs* treated cells) are shown in the columns for each experimental condition. (**B**) Migration rate in HTZ-349 cells detected with a scratch that was created in a confluent monolayer of cells. Migration rate was quantified by measuring the distance between the edges of wound and compared at *t*=0 to *t*=24 h. The migration rate is represented as percentages in graphics illustrating the increase in glioma migration with TGF-*β*2 as compared with untreated control (^**^P<0.01). Untreated (control) was expressed as 100%. Images taken after 24 h (*t*=24) incubation are shown in the upper panel. The scale bar is 200 *μ*m. *P*-values (untreated *vs* treated cells) are shown in the columns for each experimental condition. (**C**) The effect of TGF-*β*2-specific antisense PTO (AS-11) on glioma migration was analysed in HTZ-349 spheroid assays. The spheroids were allowed to migrate in absence or presence of AS-11 (20 *μ*M) and NS-mis (20 *μ*M) was used as control oligodeoxynucleotide. Results are the mean±standard deviation of two experiments. Migration areas measured as described in methods are represented in percentages. Untreated control was set to 100%.

Next, we examined the effect of TGF-*β*2 on glioma migration. We generated tumour cell spheroids from the HTZ-349 cell line and allowed the cells to migrate from the spheroids in the absence or presence of TGF-*β*2 (20 ng ml^−1^). Exposure to TGF-*β*2 significantly induced the migration rate of glioma cells in spheroid assays compared to untreated control and BSA (Figure 3A). Quantification of the migration rate in scratch assay showed significantly increased migration with TGF-*β*2 treatment, in parallel to spheroid assay results (Figure 3B; ^**^P<0.01).

### Role of versican V0/V1 in TGF-*β*2-mediated glioma migration

**Figure 4:**
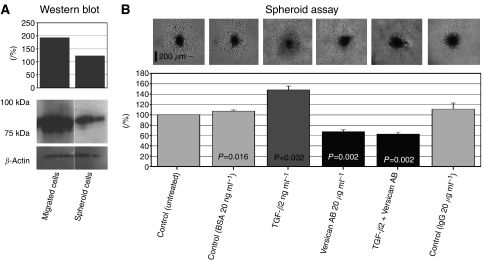
Versican modulates glioma migration mediated by TGF-*β*2. (**A**) The expression of versican at protein level was detected by Western blotting probed with versican V0/V1 antibody. Migrating cells and non-migrating spheroids were analysed separately. *β*-Actin was used as loading control. Migrating cells express remarkably higher levels of versican V1 than non-migrating cells. Densitometric values in percentages in the upper panel show almost 100% increase of versican expression in migrating cells compared to non-migrating spheroid cells. (**B**) Effect of versican on glioma migration mediated by TGF-*β*2 was analysed in spheroid assays as described in methods. The spheroids treated with TGF-*β*2 protein were also exposed to versican V0/V1 antibody (20 *μ*g ml^−1^). We also analysed spheroids exposed to versican V0/V1 antibody without TGF-*β*2. The TGF-*β*2-treated cells show significantly higher migration rates (^**^P<0.01). Versican V0/V1 antibody was able to decrease migration, both with or without exogenous TGF-*β*2 protein compared to untreated control and control IgG (^**^P<0.01). Results are shown with the mean±standard deviation of migration distance in percentages detected from two experiments; six spheroids were used for each experimental condition. Untreated (control) are expressed as 100%. Images taken at day 3 are shown in the upper panel. The scale bar is 200 *μ*m. *P*-values (untreated *vs* treated cells) are written in the columns for each experimental condition.

To elucidate the mechanisms involved in enhancement of glioma migration mediated by TGF-*β*2, we evaluated whether the upregulation of the versican isoforms V0/V1 by TGF-*β*2 might be involved in this process. We used versican V0/V1 Neo-antibody to block the GAG-*β* chain and IgG-unspecific controls. In these assays, the migration rate was significantly higher in TGF-*β*2 20 ng ml^−1^-treated spheroids compared to untreated controls (^**^P<0.01) (Figure 4B). As we detected previously that TGF-*β*2 had no effect on proliferation of HTZ-349 cells (unpublished data), proliferation is very likely to have no contribution to the increased area covered by cells migrating away from spheroids. When versican V0/V1 antibody was added to TGF-*β*2-treated spheroids, the enhancement of glioma migration by TGF-*β*2 was reversed in a dose-dependent manner. With 20 *μ*g ml^−1^ of versican V0/V1 antibody, migration was even inhibited compared to untreated control and control IgG (^**^P<0.01). We also observed similar results with versican V0/V1 antibody in the absence of exogenous TGF-*β*2. These results might explain that, even without exogenous TGF-*β*2, the anti-GAG-*β* antibody interferes with the interaction of versican with endogenous TGF-*β*2 and inhibits glioma migration, while HTZ-349 expresses and secretes high levels of TGF-*β*2 (Figures 1A and 2). AS-11-treated cells showed significant decreased migration rate (28.6%) in comparison to untreated control (100%) and control oligonucleotide (87.2%) (*P*<0.001) (Figure 3C).

We also detected that cells migrating from spheroids express significantly higher levels of versican V1 compared to immobile glioma cells within spheroids (Figure 4A) which further supports the involvement of versican in glioma migration.

## DISCUSSION

In the present study, we demonstrate marked differences in the expression patterns of versican isoforms in high-grade gliomas. The largest splice variants of versican, V0 and V1, are the predominant forms present in most glioma cell lines, whereas V2 is rarely expressed, consistent with previous studies concerning human glioma cell lines (Dours-Zimmermann and Zimmermann, 1994; Bouterfa *et al*, 1999). Expression of V0/V1 isoforms increases in different tumours (Touab *et al*, 2002), suggesting that these isoforms may be involved in tumour development. In this context, our results show that V0/V1 are the main versican isoforms related to the malignant phenotype of glioma *in vitro*. Additionally, we have demonstrated for the first time in high-grade gliomas that TGF-*β*2 is able to upregulate versican expression in a concentration- and time-dependent manner. When the expression of versican isoforms was determined with specific primers, mainly V1 was found to contribute to this upregulation, whereas V2 expression was not induced. This observation is consistent with previous reports that demonstrated that versican isoforms V1 and V2 are not only differentially expressed, but also play distinct roles in cell function which are mediated by GAG-*α* and GAG-*β* domains, respectively (Wu *et al*, 2004b; Sheng *et al*, 2005). The balanced expression of these two isoforms might provide a suitable extracellular environment for normal proliferation and survival of cells. The extracellular environment might become favourable for cell proliferation and survival when V1 expression is increased, as in the case of tissue development and tumour formation (Sheng *et al*, 2005). Recently, in parallel to our results in high-grade gliomas, TGF-*β*2 was reported to trigger the expression of V0/V1 and hyaluronan in osteosarcoma cells (Nikitovic *et al*, 2006).

There is increasing evidence for a prominent role of TGF-*β*2 in glioma cell motility. TGF-*β*2 is known to induce a malignant phenotype in glioma cell lines using exogenous TGF-*β*2 (Platten *et al*, 2001). Brockmann *et al* (2003) detected motogenic effects of TGF-*β*2 in glioblastoma cell lines and our results demonstrate that exogenous TGF-*β*2 induces migration of glioma cells significantly via two different migration assays. Moreover, TGF-*β*2-specific phosporothioate antisense oligonucleotides as described previously (Nickl-Jockschat *et al*, 2007) significantly inhibit migration compared to a control mismatch oligonucleotide in our migration assay (Figure 3C).

It is known that cancer cells' survival and motility are dependent on TGF-*β*-mediated autocrine mechanisms (Dumont and Arteaga 2003). Treatment with paracrine/exogenous TGF-*β* at higher concentrations than autocrine TGF-*β* further enhances the expression of promigratory molecules and cancer cell invasion (Shiou *et al*, 2006). We previously detected that expression of K-ras, a brain-specific isoform of Ras and the most prominent oncogene of the MAPK pathway (Spandidos and Kerr, 1998; Johnson *et al*, 2001), was downregulated exclusively in the AS-11-treated populations of glioma cell lines (Nickl-Jockschat *et al*, 2007). Ras leads, via activation of its downstream substrates, to an enhanced transcription of ECM proteins and to cytoskeletal rearrangement, favouring invasion and migration of malignant cells (Derynck and Zhang, 2003). Considering this, downregulation of K-ras might explain the inhibition of migration with AS-11 in our assays. Taken together, our results, consistent with previous reports, suggest the involvement of both autocrine and paracrine TGF-*β* mechanisms in high-grade glioma migration.

Versican-rich extracellular matrices exert an anti-adhesive effect on the tumour cells (Yamagata and Kimata, 1994; Touab *et al*, 2002), thus facilitating tumour cell migration and invasion. It has been found to be co-localised with hyaluronan, CD44 and tenascin in the pericellular matrix in tumours and because of its ability to interact with modulators of glioma migration and invasion, such as hyaluronan, tenascin, CD44, integrins and epidermal growth factor receptor (EGFR) (Wu *et al*, 2005), versican may contribute to the malignant properties of glioma cells.

We hypothesised that versican V1 overexpression induced by TGF-*β*2 might be one of the mechanisms by which TGF-*β*2 exerts invasiveness of high-grade gliomas. Versican isoforms V0 and V1 are overexpressed in tumours (Touab *et al*, 2002), suggesting that these isoforms are mainly involved in tumour development. Versican-rich extracellular matrices exert an anti-adhesive effect on tumour cells (Yamagata and Kimata, 1994; Touab *et al*, 2002), thus facilitating tumour cell migration and invasion. Versican has been found to be co-localised with hyaluronan, CD44 and tenascin in the pericellular matrix in tumours, and because of its ability to interact these modulators of invasion and EGFR (Wu *et al*, 2005), versican may contribute to the malignant properties of glioma cells. Mechanistically, versican enhances the locomotion of astrocytoma cells and reduces cell adhesion (Ang *et al*, 1999).

To understand the importance of versican V1 and TGF-*β*2 in glioma migration, we blocked functionally the GAG-*β* domain of versican with a specific antibody. Blockage of the GAG-*β* domain was able to reverse the effect of TGF-*β*2 on glioma migration in a dose-dependent manner (Figure 4B). Without exogenous TGF-*β*2, the anti-GAG domain antibody itself somehow decreased the migration rate compared to untreated cells. This effect may be due to inhibition of the interaction between versican and endogenous TGF-*β*2 secreted by HTZ-349 cells (Figure 1A). We have also found that migrating glioma cells express significantly increased levels of V0/V1 isoforms compared to non-migrating cells. These results underline the importance of versican for glioma cells during migration.

The G3 domain of versican induces glioma cell adhesion through EGFR and *β*1-integrin-mediated pathways (Wu *et al*, 2002). GAG-*β* domains somehow interfere with the G3/EGFR interaction and decrease the antiproliferative effect of G3 in melanoma cells (Serra *et al*, 2005). We speculate that blockage of this interference by a GAG-*β* domain-specific antibody in our migration assays probably induced adhesion and consecutively decreased the migration of glioma cells.

Here, we demonstrated in immunochemical analyses that exogenous TGF-*β*2 induces not only versican V1 expression, but also cleavage of versican V1 probably mediated via ADAMTS-1 and ADAMTS-4 at the Glu441–Ala442 bond in the V1 isoform; the corresponding peptide bond is Glu1428–Ala1429 in the V0 isoform (Sandy *et al*, 2001). We have also shown that TGF-*β*2 is capable of increasing MMP-2 activity and thereby induces the degradation of versican V1 (Arslan *et al*, 2006). Cleavage of brevican, another member of the lectican family by ADAMTS-5 is functionally involved in glioma invasion *in vivo* (Nakada *et al*, 2005). There is a strong likelihood that breakdown products of versican will also have biological activity in glioma probably paving the way for the invasion into tissue (Zheng *et al*, 2004). However, it is not entirely clear if proteolytic degradation of versican by MMP-2 and ADAMTS-1 and 4 induced by TGF-*β*2 has a pathophysiological role in glioma progression.

Versican appears not only to present or recruit molecules to the cell surface, but also modulates the expression levels of genes and co-ordinates complex signaling pathways. Versican V1 induces integrin-mediated extracellular signal-regulated kinase (ERK) pathway (Wu *et al*, 2004b); recently, we reported that the ERK pathway is responsible for TGF-*β* tumour promoting effects in high-grade gliomas (Nickl-Jockschat *et al*, 2007). Complex interactions of functional TGF-*β* and EGF signal cascades in human gliomas have also been described (Held-Feindt *et al*, 2003). It is currently under investigation whether versican V1 and TGF-*β*2 can interact with each other also in regard of their activated signaling cascades, such as the EGFR- and integrin-mediated ERK pathways, enhancing the malignant progression of glioma.

In conclusion, our data provide the first evidence for the functional importance of versican isoforms V0/V1 in glioma migration mediated by TGF-*β*2. Previously, the versican G3 domain was found to be important in astrocytoma cell proliferation, glioma adhesion, tumour growth and angiogenesis (Wu *et al*, 2002, 2004a; Zheng *et al*, 2004). Our results indicate that the V0/V1 isoforms modulate glioma migration through their common GAG-*β*-domain. Thus, there is mounting evidence for a crucial role of different domains of versican in glioma tumorigenesis. Since certain domains of versican possess unique biological activities *in vitro*, further studies are required to precisely define the molecular mechanisms behind the effects of such domains (isoforms) and to outline the biological consequences *in vivo*.
